# Land- and water-based exercise intervention in women with fibromyalgia: the *al-andalus *physical activity randomised controlled trial

**DOI:** 10.1186/1471-2474-13-18

**Published:** 2012-02-15

**Authors:** Ana Carbonell-Baeza, Jonatan R Ruiz, Virginia A Aparicio, Francisco B Ortega, Diego Munguía-Izquierdo, Inmaculada C Álvarez-Gallardo, Víctor Segura-Jiménez, Daniel Camiletti-Moirón, Alejandro Romero, Fernando Estévez-López, Blanca Samos, Antonio J Casimiro, Ángela Sierra, Pedro A Latorre, Manuel Pulido-Martos, Pedro Femia, Isaac J Pérez-López, Palma Chillón, María J Girela-Rejón, Pablo Tercedor, Alejandro Lucía, Manuel Delgado-Fernández

**Affiliations:** 1 Department of Physical Education and Sport, School of Sport Sciences, University of Granada, Granada, Spain; 2 Department of Physical Education, School of Education, University of Cadiz, Cadiz, Spain; 3 Department of Biosciences and Nutrition at NOVUM, Unit for Preventive Nutrition, Karolinska Institutet, Huddinge, Sweden; 4 Department of Physiology, School of Pharmacy, University of Granada, Granada, Spain; 5 Deparment of Physical Education and Sports, University Pablo de Olavide, Seville, Spain; 6 Department of Didactics for Language and Literature, Social Science and Physical and Sports Education, University of Almeria, Almeria, Spain; 7 Department of Physical Education, Music and Arts, University of Huelva, Huelva, Spain; 8 Department of Didactics of Music, Plastic and Corporal Expression, University of Jaen, Jaen, Spain; 9 Department of Psychology, University of Jaen, Jaen, Spain; 10 Biostatistics, School of Medicine, University of Granada, Granada, Spain; 11 Department of Biomedicine, European University of Madrid, Madrid, Spain

## Abstract

**Background:**

The *al-Andalus *physical activity intervention study is a randomised control trial to investigate the effectiveness of a land- and water-based exercise intervention for reducing the overall impact of fibromyalgia (primary outcome), and for improving tenderness and pain-related measures, body composition, functional capacity, physical activity and sedentary behaviour, fatigue, sleep quality, health-related quality of life, and cognitive function (secondary outcomes) in women with fibromyalgia.

**Methods/Design:**

One hundred eighty women with fibromyalgia (age range: 35-65 years) will be recruited from local associations of fibromyalgia patients in *Andalucía *(Southern Spain). Patients will be randomly assigned to a usual care (control) group (n = 60), a water-based exercise intervention group (n = 60) or a land-based exercise intervention group (n = 60). Participants in the usual care group will receive general physical activity guidelines and participants allocated in the intervention groups will attend three non-consecutive training sessions (60 min each) per week during 24 weeks. Both exercise interventions will consist of aerobic, muscular strength and flexibility exercises. We will also study the effect of a detraining period (i.e., 12 weeks with no exercise intervention) on the studied variables.

**Discussion:**

Our study attempts to reduce the impact of fibromyalgia and improve patients' health status by implementing two types of exercise interventions. Results from this study will help to assess the efficacy of exercise interventions for the treatment of fibromyalgia. If the interventions would be effective, this study will provide low-cost and feasible alternatives for health professionals in the management of fibromyalgia. Results from the *al-Andalus *physical activity intervention will help to better understand the potential of regular physical activity for improving the well-being of women with fibromyalgia.

**Trial registration:**

ClinicalTrials.gov ID: NCT01490281

## Background

Fibromyalgia is becoming a common syndrome in Western European countries since a point prevalence of 2.9% would translate to approximately 6 million people with fibromyalgia [1]. The prevalence of fibromyalgia in Spain is ~2.4%, and is more common in women (~4.2%) than in men (~0.2%) [2].

Fibromyalgia is considered a disorder of pain regulation [3], as indicated by an increased sensitivity to painful stimuli (hyperalgesia) and lowered pain threshold (allodynia) [4]. Additionally to pain, fibromyalgia symptoms typically include fatigue, stiffness, non-restorative sleep patterns, and memory and cognitive difficulties [5-7]. Other common symptoms are low back pain, recurrent headaches, muscle-spasm, and balance problems [5]. The prevalence of comorbidities among patients diagnosed with fibromyalgia is very high [8], which increases patients' needs for appropriate medical management and results in higher healthcare resource utilization compared with persons without fibromyalgia [9].

Fibromyalgia has an important impact on the patients' health-related quality of life [10,11], since it limits activities of daily life such as walking or raising and transporting objects [11,12]. In general, functional capacity is decreased in adults with fibromyalgia [13-18] and is similar to that of healthy elderly [16,19]. Jones et al. [19] reported that women with fibromyalgia have difficulties for staying physically independent.

Exercise therapy is relatively economical, easily accessible and widely used in clinical practice as a strategy for pain management. Several reviews concerning the effect of exercise in fibromyalgia patients concluded that: i) there is moderate evidence that aerobic exercise produces important benefits in physical function, pain and tender points [20,21]; ii) there is limited evidence that strength training improves pain, global wellbeing, physical function, tender points and depression [22]; and iii) there is not enough evidence regarding the health-related effects of flexibility exercises [22]. A recent meta-analysis [23] concluded that exercise (aerobic, strength training or both) improves global well-being, assessed by the fibromyalgia impact questionnaire (FIQ) in women with fibromyalgia. The Ottawa Panel supports the use of aerobic exercise interventions and strengthening exercises for the overall management of fibromyalgia [24,25].

Exercise therapy in fibromyalgia patients has usually focused on either pool or land-based exercises. A recent meta-analysis [26] indicated that there is no evidence that water-based aerobic exercise produces superior results compared to similarly intense land-based exercise. Others narrative reviews however suggested slight additional benefits for water-based aerobic exercise on reducing pain and improving depressive symptoms [27], sleep quality and mood [20]. Jentoff et al. [28] compared the effect of a 20-week pool-based exercise and a land-based exercise intervention (twice a week) on symptoms, self-efficacy, self-reported physical impairment, and physical capacity in a group of fibromyalgia patients. They observed significant improvements in cardiovascular capacity and walking time in both exercise groups. The pool-exercise group also improved self-reported physical impairment, number of days feeling good, pain, anxiety, and depression. To our knowledge, the study by Jentoff et al. [28] is the only available study investigating the possible additional beneficial effects of exercising in a warm-water pool (compared to land-based) in fibromyalgia patients. More studies comparing the effectives of a water-based exercise program with a land-based exercise intervention on a broader range of physical and psychological outcomes and with longer intervention period, higher frequency, and larger samples are needed.

It is of public health and clinical relevance to better understand whether the benefits of land-based exercise are similar to those observed with exercise undertaken in water, owing to the low availability of pools with an appropriate water temperature for these patients, ideally between 30 and 33°C.

### Objectives

The primary objective of the *al-Andalus *physical activity randomised controlled trial (RCT) is to investigate the effectiveness of two types of exercise interventions (water- and land-based aerobic-strength training vs. a control group) on the overall impact of fibromyalgia (primary outcome), and on tenderness and pain-related measures, body composition, functional capacity, physical activity and sedentary behaviour, fatigue, sleep quality, health-related quality of life and cognitive function (secondary outcomes) in women aged 35-65 years with fibromyalgia. The intervention groups will train 3 days/week (60 min per session) for a 24-week period. We will also study the effect of a detraining period (i.e., 12 weeks with no exercise intervention) on the studied variables.

## Presentation of the hypothesis

We hypothesised that: (i) a 24-weeks exercise intervention training (either water- or land-based) would be effective for reducing the impact of fibromyalgia (primary outcome), and improving tenderness and pain-related measures, body composition, functional capacity, physical activity and sedentary behaviour, fatigue, sleep quality, health-related quality of life and cognitive function (secondary outcomes) in women with fibromyalgia; (ii) a 24-weeks land-based aerobic-strength intervention training could be as effective as a water-based intervention; and (iii) the exercise training-induced gains would be partially retained in the intervention groups after the detraining period such that their physical status will still be better than that of the control group at this time point.

## Testing the hypothesis

The present study is a RCT (ClinicalTrials.gov ID: NCT01490281). The Medical Ethics Committee of *Hospital Virgen de las Nieves *(Granada, Spain) approved the study design, study protocols and informed consent procedure. All participants have to provide a written informed consent. After baseline measurements, they will be randomly allocated to the usual care (control), land-based training or water-based group. The participants will be followed for 24 weeks during the training interventions and after 12 weeks of training cessation. All the baseline and follow-up examinations will be performed in the same setting (local association of fibromyalgia patients) and by the same investigators. The study will be performed following the ethical guidelines of the Declaration of Helsinki, last modified in 2000.

### Participants and selection criteria

Women with fibromyalgia will be recruited from the local associations of fibromyalgia patients in *Andalucía *(Southern Spain). Before starting the study, a screening will be performed of all candidates. The inclusion and exclusion criteria for the study are shown in Table 1.

**Table 1:** Inclusion and exclusion criteria in the *al-Andalus *physical activity trial

*Inclusion criteria*	*Exclusion criteria*
- Age: 35-65 years.	- Acute or terminal illness.

- To be diagnosed with fibromyalgia by a rheumatologist and meeting the American College of Rheumatology criteria: widespread pain for more than 3 months, and pain with 4 kg/cm of pressure reported for 11 or more of 18 tender points [6].	- Myocardial infarction in the past 3 months.

- Not to have other severe somatic or psychiatric disorders, or other diseases that prevent physical loading (Answer "no" to all questions on the Physical Activity Readiness Questionnaire-PAR-Q [29]).	- Unstable cardiovascular disease or other medical condition.

- Not to be engaged in regular physical activity > 20 min on > 3 days/week in the past 3 months.	- Upper or lower extremity fracture in the past 3 months.

- Planning to stay in the same Association during the study.	- Unwillingness to either complete the study requirements or to be randomised into control or training group.

- Able to ambulate without assistance.	- Severe dementia (MMSE < 10)

- Able to communicate.	- Presence of neuromuscular disease or drugs affecting neuromuscular function.

- Informed consent: Must be capable and willing to provide consent.	- To be engaged in other physical or psychological treatment.

### Sample size

**Figure 1:**
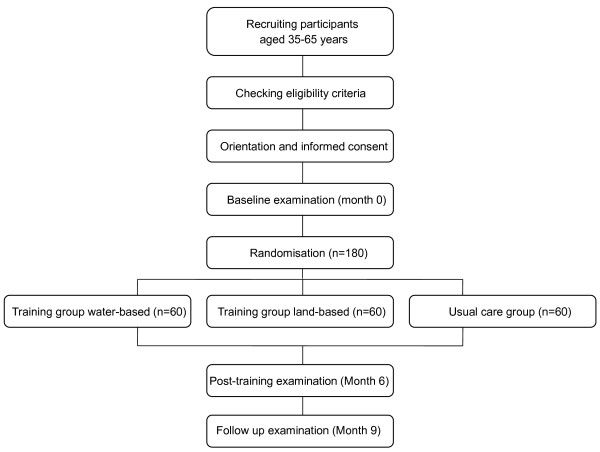
**Flow diagram of the study participants**.

The required sample size was determined for the primary outcome variable, i.e. overall score of Fibromyalgia Impact Questionnaire (FIQ) [30]. According to previous research [31], a clinically relevant change is a 15-20% reduction in the total FIQ score (which equals to a ~10-15 points reduction). Assuming an unilateral alternative (i.e. the intervention reduces impact of fibromyalgia), we can detect differences of at least 15% with a power of 95% and α of 0.05 with two groups (intervention and usual care group) of 45 participants, with a mean in the FIQ of ~70 and a standard deviation of ~20 points. Assuming a maximum lost of follow-up of 30%, we will recruit a total of 60 women with fibromyalgia for each group. Since we will develop two types of interventions (land and water-based), we will recruit a total of 180 women with fibromyalgia (i.e., two intervention groups and one usual care group of 60 persons each). Figure 1 shows the flow diagram of the study participants

### Randomisation and blinding

The participant randomisation assignment will follow an allocation concealment process, that is, the researcher in charge of randomly assigning participants will not know in advance which treatment the next person would receive and will not participate in assessment. Assessment staff will be blinded to participant randomisation assignment. Participants will be explicitly informed on the group to which they will be assigned as well as on the study hypotheses and will be reminded not to discuss their randomisation assignments with assessment staff. It will not be possible to conceal the group assignment from the staff involved in the training.

### Statistical analysis

For between groups comparisons at baseline (usual care vs water-based intervention vs land-based intervention), we will analyse continuous variables with one-way analysis of variance or the non-parametric method of Kruskall-Wallis, and Chi-square tests (or exact techniques if needed). We will use analysis of covariance (ANCOVA) to assess the training effects on the primary and secondary study outcomes (or the rank-transform ANCOVA). Age and baseline level of each outcome variable will be entered as covariates. For each outcome variable we will report the effect size and the level of significance corresponding to the main group (between-subjects), time (within-subjects) and interaction (group × time) effects.

We will adjust multiple comparisons for mass significance [32]. We will also examine the differences between drop-outs and participants who remain in the study. We will analyse the data according to the intention-to-treat principle [33]. We will handle missing data due to drop-outs or non-compliance using multiple imputation methods. To fully appreciate the potential influence of missing responses, we will perform sensitivity analysis. In all analyses, we will study the effect of the regression towards the mean.

### Interventions

The interventions will be performed in waves so that each wave will have between 10 and 12 participants in the intervention groups, and 10-12 in the usual care group. The land-based and water-based intervention groups will train 3 days/week (60 min per session) for a 24-week period. The intervention will involve exercises for improving cardiovascular endurance, muscle strength, and joint range of motion. The exercise interventions will meet the minimum training standards of the American College of Sports Medicine [34]. The exercise sessions will be carefully supervised by an exercise monitor.

The exercises will involve all major muscle groups. During the first weeks, the main part of the sessions will focus on teaching the patients how to perform the exercises. Each session will include 10 min of warm-up with slow walks and mobility exercises, followed by 35-40 min of aerobic exercises, developed progressively at intensity sufficient to achieve 50% (at the beginning of the intervention) and ~80% (the last month of the intervention) of predicted maximum heart rate (209-0.73 × age), and resistance strength training. Finally, each session will end with 10 min of cooling down with low-intensity, flexibility and relaxation exercises.

Heart rate will be assessed with a heart rate monitor (Polar Electro OY, Finland) in order to control the intensity of the sessions. One third of the patients in the intervention group will wear heart rate monitors in 1/3 of the sessions, both randomly selected. We will also monitor the rate of perceived exertion (RPE) using the Borg's conventional (6-20 point) scale [35]. Intensity (expressed as RPE) will range from 12 to 14. These RPE values correspond to a subjective perceived exertion of "light" and "somewhat hard" respectively. Overexertion will be checked by the "Talk test", that is, during exercise sessions participants will be able to maintain a conversation without getting breathlessness [36].

#### Land-based intervention

The land-based exercise intervention group will train in the exercise training facilities of the local association of fibromyalgia, or in the closer exercise centre. Cardiovascular exercises will incorporate walking at different speeds and continuous and rhythmic activities that affects large muscle groups and aerobic dance. Resistance strength training will include 1-3 set of 8-12 repetitions. The strengthening exercises will include biceps curls, arm extensions, arm side lifts, shoulder elevations, lateral leg elevations, stands up from seated position, lunge, sideways lunge and step-up/step-down. The load will be gradually increased as the strength of each person improves. We will use body weight at the start of the program and barbells (0.5-2 kg per exercise). Flexibility will be developed by static stretching at the end of the warm-up and cool-down periods.

#### Water-based intervention

The water-based exercise intervention group will train in a chest-high warm (~30°C) pool. A modified version of the land-based intervention, adapted to the restrictions and peculiarities imposed by water, will be used. The training intensity and the muscle groups activated will be as similar as possible in the two groups.

The cardiovascular exercises will include bicycling simulation (with the floater between legs), walking around the pool, continuous and rhythmic activities that affects large muscle groups and aerobic dance. The strength exercises will be performed at slow pace using water and aquatic materials as resistance. Flexibility exercises will consist of static stretching at the end of the warm-up and cool-down, as in the land-based intervention.

#### Usual care group (control)

Participants randomly assigned to the usual care (control) group will receive general advices from the exercise monitor about the positive effects of physical activity. We will prepare informative pamphlets describing the benefits of physical activity and general guidelines about how to increase the daily physical activity levels.

### Participant retention and adherence

To maximize adherence, several strategies will be implemented including music in all sessions, individualized attention at the intervention sessions, telephone calls following missed sessions, and the control of patients' pain rate before and after each session. Make-up sessions will be allowed in case of missing sessions (due to illness or any other reason).

### Primary outcomes measures

*Fibromyalgia Impact Questionnaire *(FIQ) is a self-administered questionnaire, comprising 10 subscales of disabilities and symptoms (physical function, work missed day, job ability, feel good, pain, fatigue, sleep, stiffness, anxiety and depression) and has been validated for Spanish fibromyalgia patients [30]. The total scores range from 0 to 100, with a higher score indicating greater effect of the condition on the person's life.

### Secondary outcomes measures

#### Tenderness and pain related measures

We will assess 18 *tender points *according to the American College of Rheumatology criteria for classification of fibromyalgia using a standard pressure algometer (FPK 20; Effegi, Alfonsine, Italy). The *tender point count*, total count of positive tender points, will be recorded for each participant. The *algometer score *will be calculated as the sum of the minimum pain-pressure values obtained for each tender point.

*The Visual analogic scale for pain *is a simple assessment tool consisting of a 10 cm line with 0 on one end, representing no pain, and 10 on the other, representing the worst pain ever experienced, which a patient marks to indicate the severity of her pain in the present moment. This scale will also be administered before and after each session during the intervention, to assess the acute effect of exercise on pain.

*The Pain Catastrophizing Scale *[37,38] will be used to assess three factors: rumination, magnification and helplessness associated to pain. It includes 13 items measured on a 5-point Likert scale ranging from 0 (not at all) to 4 (all the time). Higher scores indicate a greater tendency to catastrophize pain symptoms.

*The Chronic Pain Self-Efficacy Scale *[39,40], is a scale that measures efficacy expectations for coping with pain. It contains 22 items grouped into 3 subscales: self efficacy for pain management, self efficacy for coping with symptoms, and self efficacy for physical function. The scores are obtained by means of a Likert scale with a range of 0-10, where higher scores indicate better self efficacy.

#### Body composition

Weight and height will be measured, body mass index [weight (kg)/height(m^2^)] will be calculated, and skeletal muscle mass, total body water and fat free mass will be estimated with bioelectrical impedance analysis (InBody R20; Biospace, Gateshead, UK).

#### Functional capacity

Participants' functional capacity will be assessed by field-based fitness tests following the standardised Functional Senior Fitness Test Battery [41]. This battery assesses muscular strength, flexibility, balance and aerobic capacity by means of the following tests:

##### Lower body muscular strength

The "30-s chair stand test" involves counting the number of times within 30 s that an individual can rise to a full stand from a seated position with back straight and feet flat on the floor, without pushing off with the arms. The patients will perform one trial after familiarization [41].

##### Upper body muscular strength

The "Arm curl test" involves determining a number of times a hand weight (2.3 kg for women) can be curled through a full range of motion in 30 s [41]. Additionally, handgrip strength will be measured using a digital dynamometer (TKK 5101 Grip-D; Takey, Tokyo, Japan) as described elsewhere [42]. Patients will perform (alternately with both hands) the test twice allowing a 1-min rest period between measures. The best value of 2 trials for each hand will be chosen and the average of both hands will be used in the analyses.

##### Lower body flexibility

In the "chair sit and reach test", the patient seated with one leg extended, slowly bends forward sliding the hands down the extended leg in an attempt to touch (or pass) the toes. The number of centimeters short of reaching the toe (minus score) or reaching beyond it (plus score) will be recorded [41]. Two trials with each leg will be measured and the best value of each leg will be registered, and the average of both legs will be used in the analyses.

##### Upper body flexibility

The "back scratch test", a measure of overall shoulder range of motion, involves measuring the distance between (or overlap of) the middle fingers behind the back with a ruler [41]. Participants will perform this test twice, alternately with both hands, and the best value will be registered. The average of both hands will be used in the analyses.

##### Motor agility/dynamic balance

The "8 ft up and go test" involves standing up from a chair, walking 8 ft to and around a cone, and returning to the chair in the shortest possible time [41]. The best time of two trials will be recorded and used in the analyses.

##### Cardiorespiratory fitness

We will use the "6-min walk test". This test involves determining the maximum distance (meters) that can be walked in 6 min along a 45.7 m rectangular course [41,43].

*The International Fitness Scale *[44] is composed of five Likert-scale questions asking about the perceived patients' overall fitness, cardio-respiratory fitness, muscular fitness, speed-agility and flexibility in comparison with their friends' physical fitness ("very poor", "poor", "average", "good" and "very good").

#### Physical activity and sedentary behaviour

Physical activity and sedentary behaviour will be objectively (i.e. accelerometry) and subjectively (i.e. questionnaires) assessed:

##### Accelerometry

Women will be asked to wear an tri-axial accelerometer (ActiGraph GT3X+, Pensacola, Florida, US) for 8 consecutive days, starting the same day they receive the monitor, and will returned the accelerometers to the researcher 9 days later. Participants will be instructed to wear the accelerometer on their lower back attached by an elastic belt during waking as well as sleeping hours. For security reasons, participants will be asked to take them off while bathing.

We will exclude from the analyses bouts of 60 continuous minutes of 0 activity intensity counts, considering these periods as non-wearing time. Monitor wearing time will be calculated by subtracting the non-wear time and the sleeping time (recorded through a diary) from the total registered time for the entire day (typically 1,440 min). A recording of more than 20,000 counts per minute will be considered as a potential malfunction of the accelerometer and the value will be excluded from the analyses. The first day of recording will not be included in the analysis. A total of 7 days of recording with a minimum of ten or more hours of registration per day will be necessary to be included in the study. Physical activity levels will be shown as follows: (i) average physical activity, expressed as mean counts per minute. It is a measure of overall physical activity. We will calculate mean counts per minute by dividing the sum of total counts per epoch for a valid day by the number of minutes of wear time in that day across all valid days. We will also calculate the time engaged in light, lifestyle, moderate, and moderate and vigorous intensity physical activity based upon a standardized cut-off of 100-759, 760-1,951, 1,952-5,724, and at least 1,952 counts per minute, respectively. Sedentary time will be calculated as the amount of time accumulated below 100 counts per minute during periods of wear time.

*The Leisure time physical activity instrument *[45,46] is composed of 4 items with 3 activity levels: light, moderate, and vigorous (a short description of each category will be presented). Patients will be asked to recall the average number of hours a week during the previous 4 weeks that they had spent engaged in a particular type of physical activity and at what activity level. The scale will be simplified into the following 3 levels: (1) 0.5-1.5 h a week, (2) 2-4 h a week, and (3) more than 4 h a week, and the patient will be asked to provide answers in hours. The number of hours indicated by the patient for each intensity category will be summed to obtain the leisure time physical activity level for 1 week.

*The Physical activity at homework or workplace instrument *[45,46] is composed of 7 items with 3 categories for work performed at home (light, moderate and heavy activity) and 4 categories for employment (sedentary, light, moderate and heavy activity).The hours for each category will be summed to obtain the total score.

*The Sedentary Behavior Questionnaire *[47] consisted of reports of time spent in 9 sedentary behaviors (watching television, sitting while playing computer/video games, sitting while listening to music, sitting and talking on the phone, doing paperwork or office work, sitting and reading, playing a musical instrument, doing arts and crafts, sitting and driving/riding in a car, bus, or train).

The short version of the *ALPHA Environmental questionnaire *[48,49] will be used to assess the environmental perceptions physical activity. The questionnaire will provide information about types of residences in the neighborhood, distances to local facilities, walking or cycle infrastructure in the neighborhood, cycling and walking network, neighborhood safety, home and work/study environment mode of active travel.

#### Fatigue

*The Multidimensional Fatigue Inventory *[50], that will be used to measure fatigue severity, comprises five subscales: general fatigue, physical fatigue, mental fatigue, reduced activity, and reduced motivation [2]. Each subscale includes four items with five-point Likert scales. General fatigue includes general statements about fatigue and decreased functioning and is designed to encompass both physical and psychological aspects of fatigue. Physical fatigue concerns physical sensations related to fatigue. Mental fatigue pertains to cognitive functioning, including difficulty concentrating. Reduced activity refers to the influence of physical and psychological factors on the level of activity. Reduced motivation relates to lack of motivation for starting any activity. Scores on each subscale range from 4 to 20, with higher scores indicating greater fatigue.

#### Sleep quality

*The Pittsburgh Sleep Quality Index *[51] will be used to assess sleep quality and disturbances over a l-month time interval. Nineteen individual items generate seven "component" scores: subjective sleep quality, sleep latency, sleep duration, habitual sleep efficiency, sleep disturbances, use of sleeping medication, and daytime dysfunction. The sum of scores for these seven components yields one global score. This test has been used in previous studies in fibromyalgia patients [52,53].

#### Health-related quality of life

We will determine patients' quality of life with the *Short-Form Health Survey 36 *(SF-36) [54]. The SF-36 is a generic instrument for assessing health-related quality of life. It contains 36 items grouped into 8 dimensions: physical functioning, physical role, body pain, general health, vitality, social functioning, emotional role, and mental health. The scores range from 0 to 100 in every dimension, where higher scores indicate better health.

*The Beck Depression Inventory-II *[55,56] will be used to assess depression severity. It contains 21 items and the range of score is 0-63 with higher score indicating greater depression.

*The State Trait Anxiety Inventory-I *[57] will be used to assess the level of current anxiety. It is a 20-item self-administered questionnaire; the range of score is 20-80, with higher scores indicating a greater state of anxiety.

Positive health will be assessed by means of the following questionnaire:

*The Trait Meta-Mood Scale *[58], is comprised of three subscales and we will use only one of them, the mood repair scale (8 items), that assesses how well individuals regulate their moods and repair negative emotional experiences (e.g. "when I become upset, I remind myself of all the pleasures in life"). Participants rate their responses using a 5-point Likert type scale, with 1 = "strongly disagree" to 5 = "strongly agree".

*The Positive and Negative Affectivity Schedule *[59,60] is a 20-item questionnaire designed to measure positive and negative affectivity. The questionnaire includes 10 positive and 10 negative emotional states that should be answered on a 5-point Likert scale. The negative and positive subscales represent two relatively independent scales.

*The Satisfaction With Life Scale *[61,62] designed to assess global life satisfaction. The SWLS consists of five items with a 7-point Likert-scale from "strongly disagree" to "strongly agree". Higher scores reflect greater subjective well-being.

*The Life Orientation Test Revised *[63,64], is a 10-item scale that assesses subjects' expectations about their future and their general sense of optimism. It is comprised of 3 positively worded (e.g., "I'm always optimistic about my future"), 3 negatively worded items (e.g. "I hardly ever expect things to go my way") and the other 4 are filler questions. Each item is rated on a five-point Likert type scale ranging from one ("strongly disagree") to five ("strongly agree"). The higher the score obtained in the test, the higher the level of dispositional optimism is and vice-versa.

#### Cognitive function

*The Mini Mental State Examination *(MMSE) [65] will be used to evaluate cognitive capacity and severity of dementia for the exclusion criteria. The MMSE is a brief cognitive screening test. The MMSE asks questions that assess five areas of cognitive functioning: orientation, immediate memory, attention/concentration, delayed recall and language.

*The Paced Auditory Serial Addition Task *[66] will be used to measure sustained and divided attention, auditory information processing speed, and stimulus competition filtering skill. In this study, it will be administered only at the slowest presentation rate of 2.4 s. The score is the number of correct responses over 60 trials.

*The Rey Auditory Verbal Learning Test *[67] measures immediate free recall, delayed free recall, delayed recognition, and verbal learning. This is a multiple-trial verbal list learning test and will be given on an individual basis.

#### Patient global impression of change

We will also assess the patients' perception of change after the exercise interventions with *Patient Global Impression of Improvement scale *[68]. This is a scale used by patients to report their overall assessment of change during a clinical trial that uses a 7-point rating scale with the options "very much improved", "much improved", "minimally improved", "no change", "minimally worse", "much worse" and "very much worse".

### Assessment

The outcomes will be assessed just before the intervention (baseline), after 24 weeks of exercise interventions (post-intervention) and after 12 weeks of training cessation (detraining). The assessments of the primary and secondary outcomes will be performed on two separate days in order to prevent fatigue in the patients. The patients will complete some questionnaires at home between day 1 and 2. The study assessment schedule is shown in Table 2.

**Table 2:** Study Assessment Schedule

Assessment	Screening	Baseline	Intervention	Post-intervention (24 weeks)	Detraining (12 weeks)
- Informed Consent	X				

*Day 1 testing*					
- Sociodemographic data	X			X	X
- Mini Mental State Examination	X				
- Physical Activity Readiness Questionnaire-PAR-Q	X				
- Beck Depression Inventory-II	X			X	X
- Tender points	X			X	X
- Visual analogic scale for pain		X	X	X	X

*Questionnaires to complete at home between day 1 and 2*					
- Fibromyalgia Impact Questionnaire		X		X	X
- Short-Form Health Survey 36		X		X	X
- State Trait Anxiety Inventory-I		X		X	X
- Trait Meta-Mood Scale		X		X	X
- Positive and Negative Affectivity Schedule		X		X	X
- Satisfaction With Life Scale		X		X	X
- Life Orientation Test Revised		X		X	X
- Pittsburgh Sleep Quality Index		X		X	X
- Multidimensional Fatigue Inventory		X		X	X
- ALPHA Environmental questionnaire		X		X	X
- Sedentary Behavior Questionnaire		X		X	X
- Physical activity at homework or workplace instrument		X		X	X
- Leisure time physical activity instrument		X		X	X
- International Fitness Scale		X		X	X
- Chronic Pain Self-Efficacy Scale		X		X	X
- Pain Catastrophizing Scale		X		X	X

*Day 2 testing*					
- Paced Auditory Serial Addition Task		X		X	X
- Rey Auditory Verbal Learning Test		X		X	X
- Body composition		X		X	X
- Chair sit and reach test		X		X	X
- Back scratch test		X		X	X
- Arm curl test		X		X	X
- 30-s chair stand test		X		X	X
- 8 ft up and go test		X		X	X
- Handgrip strength		X		X	X
- 6-min walk test		X		X	X
- Accelerometry (1 week)		X		X	X

- Patient Global Impression of Improvement scale				X	

### Familiarization and reliability assessment

Before the start of the study all patients will have a familiarization period with all the functional capacity tests, consisting of two ~50-min sessions. Each session will be preceded by a warm-up and will end with a cool-down of the same activities and duration used during the training period. Each familiarization session will consist of 2-3 sets of 1-3 repetitions of the exercises. We will also assess test-retest reliability (2-weeks apart) of the study functional capacity tests in a sub-sample. The tests will be performed by trained and qualified researchers.

### Assessment of side effects

We will record adverse effects or health problems attributable to the testing sessions or intervention sessions, including muscle pain, fatigue, and general aches and pains by self-report during the study period. An independent researcher will be in charge of auditing all assessment staff to record all these events in the participants over the study period.

## Implications of the hypothesis

The *al-Andalus *physical activity RCT will investigate the effects of 24-week land- and water-based exercise interventions on disease impact, tenderness, body composition, functional capacity, quality of life and cognitive function in female patients with fibromyalgia. Both exercise interventions will include aerobic, strengthening and flexibility exercises. We will also assess the effect of a 3-month detraining period on the study primary and secondary outcomes. To our knowledge, this is the largest study specifically designed to compare the effect of a water-based and land-based exercise interventions on fibromyalgia patients. Our study attempts to reduce impact of fibromyalgia and improve patients' health status and quality of life by implementing two types of exercise interventions. Results from this study will help to better assess the potential of exercise interventions in the treatment of fibromyalgia. If the interventions proved to be effective and safe, this study would provide low-cost and feasible alternatives for health professionals in the management of fibromyalgia.

## Abbreviations

ANCOVA: Analysis of covariance; FIQ: Fibromyalgia impact questionnaire; MMSE: Mini Mental State Examination; PAR-Q: Physical Activity Readiness Questionnaire; RCT: Randomised controlled trial; RPE: Rate of perceived exertion; SF-36: Short-form 36 items.

## Competing interests

The authors declare that they have no competing interests.

## Authors' contributions

ACB, JRR, VAA and MDF conceived the study, and participated in its design. ACB, JRR and VAA drafted the manuscript. FBO, DM, VS, AR, FE, BS, AJC, AS, PL, MP, PF, IJP, PC, MJG, PT and AL participated in the study design and critically revised the manuscript. ICA, DC and BS participated in the study design, critically revised the manuscript, supervised and design the exercise interventions. PF participated in the study design and provided statistical support. All authors read and approved the final manuscript.

## Pre-publication history

The pre-publication history for this paper can be accessed here:

http://www.biomedcentral.com/1471-2474/13/18/prepub
